# Happy birthday, Klaus-Rüdiger! Heartfelt appreciation on the occasion of the 80th birthday of Professor Klaus-Rüdiger Trott

**DOI:** 10.1007/s00066-020-01668-y

**Published:** 2020-07-16

**Authors:** Stephanie E. Combs, Michael J. Atkinson

**Affiliations:** 1 Departments of Radiation Oncology & Radiation Biology, Technische Universität München (TUM), Munich, Germany; 2 Institutes of Radiation Medicine and Radiation Biology, Helmholtz Zentrum München, Munich, Germany

With offices in Munich, London, Nurnberg, and even the Bavarian lakes. An unstoppable and indefatigable enthusiast for radiation biology, radiation oncology, and radiation protection. An empathic and supremely supportive teacher, a dedicated historian, theater and opera aficionado, caring spouse, and tireless discussant!

Professor Klaus-Rüdiger Trott will reach a milestone in July 2020—his 80th birthday. His engine still runs on high energy, with no compromise.

When Klaus-Rüdiger went to medical school in Munich, he was almost immediately drawn to radiation sciences. This led him in the late sixties to the radiation biology lab of Otto Hug at the Ludwigs-Maximilians University (LMU) in Munich and the newly formed Institute of Biology at the *Gesellschaft für Strahlenforschung* (GSF) in Neuherberg, which is now the *Zentrum für Gesundheit und Umwelt* (HMGU). He investigated the effects of radiation from the “frog perspective”, using frog skin as an early translational model. His interests soon metamorphosed into the study of the effects of radiation on single cells, a topic enjoying a major renaissance 50 years later. Over the next decade, Klaus-Rüdiger’s medical interests began to resurface and his research in the 70s began to show a decided bias towards experimental radiation oncology. A characteristic of this early era was his willingness to revolutionize knowledge in the field by challenging almost every preconceived notion by looking at problems from the clinical and biological perspective. The 80s saw a shift in emphasis, as, like many of his generation, Klaus-Rüdiger was caught up in the Chernobyl disaster. Unlike some compatriots, Klaus-Rüdiger never shied away from presenting a balanced and honest evaluation of the health consequences. It was probably this experience that fired his interest in radiation protection, cemented by years of service on the *Strahlenschutz Kommission* (SSK) of the German government. Allegedly, his avowed goal was to prevent the commission from making too many unscientific pronouncements. In reality, he was a serious and significant contributor to developing the policy for deep storage of nuclear waste, a concept that fell out of favor at the end of the century, but which, 50 years later, has returned as national policy.

During his Munich years, Klaus-Rüdiger developed a strong connection to the Dresden University Radiobiology Group through his dear friend Thomas Herrmann. This led him to forge ties with the GDR, where he was considered the “international expert” to explain all issues in German. Although politics meant that Dresden was a relative backwater in international science at this time, 50 years later, it is now a major internationally recognized player.

Research in the mid-80s focused on radiation effects on the heart, a field very contrary to textbook learning. Klaus-Rüdiger’s work challenged the concept that the heart was a radiation-resistant organ, and finally, 50 years later, this is the generally accepted theory.

Ever the anglophile, Klaus-Rüdiger finally saw the light and moved to London at the end of the 80s as Professor for Radiation Biology at St. Barts Hospital, part of the renowned University College of London (UCL). Here, he found a new home and created a successful radiobiology lab, networked within London, the United Kingdom, Europe, and the rest of the world. This is where he was finally able to act on one of his core values: the education and training of the next generation. His academic position and training allowed him to realize a dream to educate young students and postgraduates in radiation biology. At UCL, he founded the European Masters Course in Radiation Biology, sometimes called Klaus Trott’s travelling circus, because of his idea to bring the students to the teacher rather than the traditional alternative. Students were allowed to rotate across Europe, receiving very intense teaching from an international faculty of the leading European radiation researchers. Whilst some may have seen this as a test of endurance, one can only wonder at the successes. Ex-students are now positioned as leaders in the field, spread across the world, holding appointments in well-recognized institutions such as the German Cancer Research Center (DKFZ) in Heidelberg, the International Atomic Energy Agency (IAEA) in Vienna, the World Health Organization (WHO) in Geneva, as well as several European and non-European Universities and Governmental Agencies. It has been said that the Article 31 Expert Group of the European Commission is in fact a Klaus-Rüdiger-family get together.

When Klaus-Rüdiger officially retired from UCL in London, he of course continued teaching and overseeing the UCL MSc course. However, together with his lovely wife Uta-Elisabeth, he diverted most of his private life back to Munich, to the Bavarian Alps and lakes, and to his wife’s hometown of Nurnberg.

With the advent of tuition fees in the UK, it was clear that the majority of students could no longer afford the course. Klaus-Rüdiger came up with a logical, if somewhat unusual solution: move the course to home. This was realized in 2016 when the Technical University of Munich (TUM) was able to offer a 2-year master’s degree in Radiation Biology. It has taken almost 50 years to complete this circle, but through powers of persuasion, logical arguments, and a lot of very hard work, the course started teaching in 2016. Of course, one of the key lecturers is the untiring Klaus-Rüdiger Trott, who somehow manages the lion’s share of the teaching whilst still maintaining a fatherly eye over the welfare of the students, with, of course, considerable behind-the-scenes work from Uta-Elisabeth. Today, he can still be found in the labs and clinics almost on a daily basis, and the students love their knowledgeable and caring professor.

Together with his wife, Uta-Elisabeth, who serves as a continuous and critical observer of Klaus-Rüdiger’s career, and as a lector of his essays and publications, he finds some time to enjoy art and music. Never being inclined to show favoritism, Klaus and Uta have decided that the cultural offerings of Munich and London are both worthy of their attention. In a decision worthy of Solomon, they have chosen to split their cultural activities between Munich and London. Tickets to theaters, opera houses, and concert halls, as well as visits of exhibitions are an essential part of their routine. Regular visits to London are packed with visits to the Coliseum, Covent Garden, galleries, and exhibitions, as well as regular bicycle rides through Regent’s Park and the Royal Botanic Gardens. Back in Germany it is *Gasteig*, the *Staatstheatre*, and the many museums that take their interest, and the English Garden is of course the scene of the bicycle tours. Probably unknown to most is Klaus’ second academic life, where under the tutelage of Uta, they have researched, documented, catalogued, and published the family history of Uta-Elisabeth’s family. This culminated last year in a highly acclaimed exhibition in Nurnberg dedicated to the Speth-Falk-Hammersbacher family. Clearly from the hand of the Trott’s, the exhibition featured an equal mixture of art and science.

One word can be used to sum up the contribution of Klaus-Rüdiger Trott to radiation biology. That word is the Hawaiian *Ohana*. If you look this up, you will see that it means “family” as in “extended family” as in “blood-related *and* adoptive *and* international”; especially, it means “nobody will be left behind.”

